# Impact of intrafraction prostate motion on clinical target coverage in proton therapy: A simulation study of dosimetric differences in two delivery techniques

**DOI:** 10.1002/acm2.12714

**Published:** 2019-09-03

**Authors:** Zhong Su, Roelf Slopsema, Stella Flampouri, Zuofeng Li

**Affiliations:** ^1^ Department of Radiation Oncology University of Florida, and University of Florida Health Proton Therapy Institute Jacksonville FL USA

**Keywords:** double scattering, interplay effect, intrafraction motion, prostate, proton therapy, uniform scanning

## Abstract

**Purpose:**

To investigate the dosimetric impact of prostate intrafraction motion on proton double‐scattering (DS) and uniform scanning (US) treatments using electromagnetic transponder‐based prostate tracking data in simulated treatment deliveries.

**Methods:**

In proton DS delivery, the spread‐out Bragg peak (SOBP) is created almost instantaneously by the constant rotation of the range modulator. US, however, delivers each entire energy layer of the SOBP sequentially from distal to proximal direction in time, which can interplay with prostate intrafraction motion. This spatiotemporal interplay during proton treatment was simulated to evaluate its dosimetric impact. Prostate clinical target volume (CTV) dose was obtained by moving CTV through dose matrices of the energy layers according to prostate‐motion traces. Fourteen prostate intrafraction motion traces of each of 17 prostate patients were used in the simulated treatment deliveries. Both single fraction dose‐volume histograms (DVHs) and fraction‐cumulative DVHs were obtained for both 2 Gy per fraction and 7.25 Gy per fraction stereotactic body radiotherapy (SBRT).

**Results:**

The simulation results indicated that CTV dose degradation depends on the magnitude and direction of prostate intrafraction motion and is patient specific. For some individual fractions, prescription dose coverage decreased in both US and DS treatments, and hot and cold spots inside the CTV were observed in the US results. However, fraction‐cumulative CTV dose coverage showed much reduced dose degradation for both DS and US treatments for both 2 Gy per fraction and SBRT simulations.

**Conclusions:**

This study indicated that CTV dose inhomogeneity may exist for some patients with severe prostate intrafraction motion during US treatments. However, there are no statistically significant dose differences between DS and US treatment simulations. Cumulative dose of multiple‐fractions significantly reduced dose uncertainties.

## 1 INTRODUCTION

Prostate cancer is one of the most common cancers among men. When it is detected and treated at its early stage, disease control and patient survival are relatively high.^1^ Proton therapy is one of the treatment modalities used in prostate cancer radiotherapy. Sharp distal dose fall‐off and less integral dose are a couple of its intrinsic advantages.^2^ There are three commonly used proton treatment delivery techniques: double scattering (DS), uniform scanning (US), and pencil‐beam scanning (PBS).^3^, ^4^, ^5^ PBS prostate treatment is available at some of the new proton therapy institutions; DS and US deliveries are used by some proton therapy clinics. DS and US deliver the radiation dose to the target as a spread‐out Bragg peak (SOBP).^6^ PBS treatment plans are inversely optimized and can be delivered through single‐field uniform dose plans or multi‐field optimizations plans. In DS delivery, the SOBP covers the entire clinical target volume (CTV) at a given instance (0.1 s interval for IBA system). Target intrafraction motion will cause CTV dose degradation due to target movement outside the SOBP or beam’s eye view. On the other hand, in US and PBS delivery, energy layers are delivered in a distal to proximal direction; thus, there is a temporal‐spatial variation of the radiation dose inside the treated volume during the treatment process. This interplay effect of prostate intrafraction motion on PBS treatment was simulated and studied by Tang et al.^7^ In this study, the effects of prostate motion on dose delivery during DS and US treatment were simulated and studied using real patient prostate traces. The prostate CTV individual fraction dose distribution and 14‐fraction cumulative dose distribution were obtained using 17 patient intrafraction motion traces in the 2 Gy per fraction simulations. For prostate stereotactic body radiotherapy (SBRT), five fractions with 7.25 Gy per fraction were simulated; both individual fraction and five fraction cumulative dose distributions were obtained. We evaluated how prostate dose distributions are affected by the interplay between prostate motion and the energy‐layer delivery.

## 2 MATERIALS AND METHODS

### 2.1 Proton therapy delivery techniques

At the University of Florida Health Proton Therapy Institute (UFHPTI), IBA (Brussels, Belgium) proton treatment machines^8^ are used to treat radiotherapy patients of various disease sites. DS, US and PBS delivery techniques are available for patient treatment; compared to DS technique, US and PBS can treat patients with deep‐seated tumors and those who require large treatment portals.

When DS treatment is delivered, the range modulator rotates at 600 RPM. Depending on the modulation width, the proton beam current is turned on for a predetermined portion of the modulator track. Each step on the modulator track corresponds to an energy layer to be delivered. With the second scatter in the beamline to create a flat axial‐beam profile, the entire SOBP is delivered in one revolution of the range modulator, which takes 0.1 s. Thus, at a given instance, an entire stationary target can be covered by the SOBP. In US treatment delivery, the second scatter is replaced by two sets of beam scanning magnets in the two orthogonal directions perpendicular to the beam axis. Each energy layer is delivered through magnetic sweeping of the beam spot in the two directions. At UFPTI, the scanning frequency is 3 Hz in the gantry rotation axis direction and 30 Hz in its orthogonal direction. There is multiple repainting of each energy layer. Furthermore, the range modulator is static during the delivery of each energy layer and only rotates when beam is off between the deliveries of the energy layers. The energy layers are delivered sequentially from the most distal to the most proximal ones. At a given instance, either a nonuniform dose is delivered to the entire target (when the most distal layer is delivering) or only part of the target receives the radiation dose (all other layers). Using the US technique, the maximum beam range is increased to 32.4 cm and the treatable field size is increased to 30 cm by 40 cm. Therefore, the US technique can treat some deep‐seated tumors as well as extremely large targets. However, compared to DS delivery, US delivery is potentially more susceptible to the spatial‐temporal interplay due to target motion and nonuniform target dose delivery. When the target motion is in the beam direction, part of the target can be over‐irradiated by different layers, or it can be underdosed by missing irradiation from an energy layer, depending on the motion characteristics and layer‐delivery timing.^9^

### 2.2 Prostate traces

Prostate‐motion traces acquired using a Calypso electromagnetic transponder tracking system of 17 patients were obtained from the Virginia Commonwealth University Department of Radiation Oncology with institutional review board (IRB) approval. The Calypso 4D localization system localizes and tracks electromagnetic transponders implanted in the patient’s target volume. The overall system components and operating principles have been described by Balter et al.^10^ This system can measure the target position with submillimeter accuracy at a rate of 10 Hz. The intrafraction prostate traces were from patients treated with intensity‐modulated radiation therapy (IMRT). The dose fractionations were slightly different among the patients, but they ranged from 32 to 39 fractions with total doses ranging from 72 to 78 Gy. Prostate motion tracking time was different among patients as well as between different treatment fractions for the same patient and ranged from 4 to 10 min. The patient prostate motion characteristics varied from patient to patient; however, each patient exhibited some consistency in intrafraction motion characteristics among different fractions. Patient prostate motion exhibited slow drifts, transient excursions, or persistent deviations from the initial setup position.^11^ Detailed prostate intrafraction motion information can be found in reference 10. In summary, nine out of 17 patients have at least 10% of the tracked time that their prostate moved beyond 3 mm (three‐dimensional distance) from initial setup position; three out 17 patients have at least 10% of the tracked time that their prostate moved beyond 5 mm (three‐dimensional distance) from initial setup position; one out 17 patients has at least 10% of the tracked time that their prostate moved beyond 7 mm (three‐dimensional distance) from initial setup position. It was also observed that there is a relatively strong positive correlation between the prostate superior‐inferior (SI) direction motion and its anterior‐posterior (AP) motion. One of the methods to quantify the intrafraction motion is to calculate its systematic and random components from the patient cohort. The systematic component is calculated as the standard deviation of population‐average prostate displacement. The random component is calculated as the root‐mean‐square of the patient‐specific standard deviation of prostate displacement. Data analysis of the motion traces showed that the systematic components of prostate intrafraction motion were 0.3, 0.5 and 0.6 mm for left‐right (LR), SI and AP directions, respectively; the random components of prostate intrafraction motion were 0.7, 1.4 and 1.9 mm for left‐right (LR), SI and AP directions, respectively.

### 2.3 Prostate proton treatment simulation

**Figure 1:**
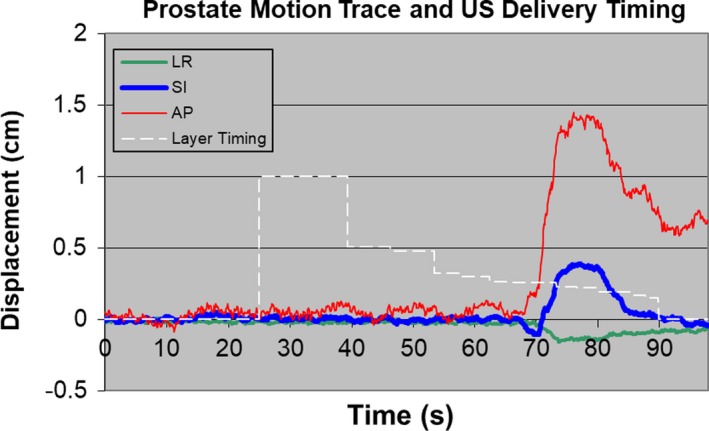
Prostate intrafraction motion trace and US energy‐layer delivery timeline. The field has a range of 30.1 g/cm^2^ and a modulation width of 8.4 g/cm^2^, which requires 14 energy layers. US, uniform scanning.

At UFPTI, left and right lateral or lateral‐oblique beams are often used to treat prostate patients.^12^ All of these patients have saline filled gas release rectal balloons inserted into their rectum in each treatment fraction. The saline filled balloons ensured no dramatic range variations posterior to the prostate and reduced prostate intrafraction motion.^13^, ^14^ For the majority of these patients, only one lateral (oblique) treatment beam is used per day, and the left and right treatment beams alternate during the course of treatment. During treatment, the left‐right prostate motion interplays with sequential energy‐layer delivery, whereas the anterior‐posterior and superior‐inferior motion will shift part of the target away from the beam’s eye view. Figure 1 shows patient intrafraction prostate motion and simulated proton energy‐layer irradiation time. MATLAB was used to simulate prostate proton treatment to evaluate the dosimetric impact of prostate intrafraction motion on proton therapy. Before the simulation, patient CT images, CTV contours, 3D dose matrices of the DS treatment plan from Eclipse treatment planning system, 3D dose matrices of each individual energy layer of the treatment beam from the US treatment plan, and prostate‐motion traces from the electromagnetic transponder system were imported into MATLAB. The prostate‐motion traces were down‐sampled from a resolution of 0.1 s to 1 s. The single fraction dose of the prostate CTV can be calculated as following:

$$D\left(CTV\left(x_{o},y_{o},z_{o}\right)\right)=\sum_{t}\left[d_{t}\left(x,y,z\right)\;\cap \;CTV\left(x_{o}\left(t\right),y_{o}\left(t\right),z_{o}\left(t\right)\right)\right].$$

where $D\left(CTV\left(x_{o},y_{o},z_{o}\right)\right)$ is the accumulated dose of a single fraction; d_t_(x,y,z) is the dose matrix at a given second; $CTV\left(x_{o}\left(t\right),y_{o}\left(t\right),z_{o}\left(t\right)\right)$ refers to the CTV voxel coordinates at a given second t based on motion traces. For DS delivery, d_t_(x,y,z) is simply the planned 3D fractional dose rescaled to a single second, whereas for US delivery, d_t_(x,y,z) is the 3D dose of the single energy layer rescaled to a single second.

During the simulation of DS treatment delivery, the prostate CTV was rigidly shifted through the dose matrix according to the prostate‐motion trace. For each second, the dose to the CTV was updated based on the current CTV location in the planned dose matrix using the motion traces. This new dose of one second was accumulated into single fraction CTV dose delivered. Before the US treatment delivery simulation, each 3D dose matrix of different energy layers was scaled using its corresponding pristine peak weight of the treatment beam. All these matrices created a four‐dimensional temporal‐spatial dose matrix such that its temporal axis was indexed by energy layer delivery time interval from the distal to the proximal end sequentially. During the simulation of US treatment delivery, using the time and location prostate‐motion trace, prostate CTV was rigidly shifted through the four‐dimensional temporal‐spatial dose matrix and the accumulated doses from all the energy layers of the treatment beam were obtained as the single fraction prostate CTV dose.

### 2.4 Prostate motion dosimetry

For patient proton treatment simulations, treatment plan data from one of the prostate patients treated with the US delivery technique at UFPTI were selected for this study. For study purposes, data from this single US patient were used in all the selected 14‐fraction prostate‐motion traces for each of the 17 patients to establish a 17‐patient group with varying intrafraction prostate motion. Using single patient geometry in the simulation allowed us to solely focus on the intrafraction motion variation and its dosimetric impact on proton treatment delivery using DS and US techniques. Otherwise, the simulation results would be more convoluted with factors such as the treatment plan differences among the patients. The prostate patient proton treatment plan included two treatment fields, the left‐anterior‐oblique (LAO) and the right‐anterior‐oblique (RAO) fields. For each treatment field, the distal, proximal and smearing margin used were 5, 10, and 19 mm, respectively. Planned target volume (PTV) expansion is 4 mm axially and 6 mm in the SI direction. Patients were treated to one treatment field per day with these two fields in an alternating order. This scheme of alternating treatment beams for each fraction was also implemented in the simulated prostate treatments with seven alternating fraction traces used for LAO and RAO each. In each simulated treatment fraction of 2 Gy per fraction, the first 91 s of prostate‐motion traces were used with the first 25 s designated as the time between finalized patient target alignment and the onset of the proton treatment. The treatment time was set at 60 s, which corresponds to a 2 Gy‐per‐minute treatment dose rate. A half second break time between the deliveries of energy layers was also simulated in the US treatment. For each fraction of SBRT simulation, the first 25 s were also designated as the time between finalized patient target alignment and the onset of the proton treatment. The treatment time was set at 224 s with 2 Gy‐per‐minute dose rate and half second layer switching time. DVH points, V100, V95, V110, D100, D95, and D5 were obtained for each fraction dose as well as their fraction cumulative for each patient.

## 3 RESULTS

**Figure 2:**
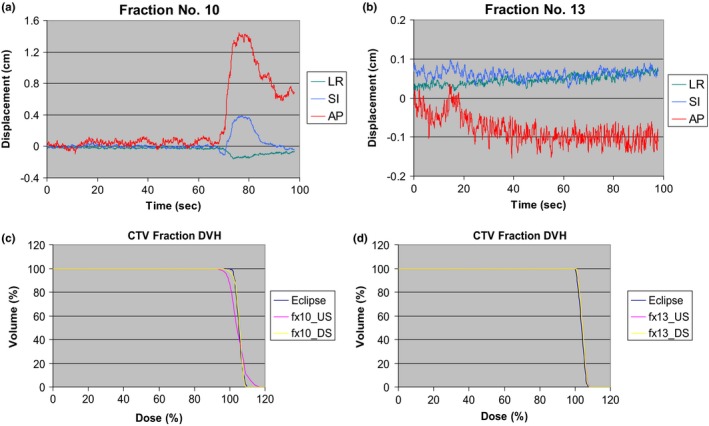
A single patient intrafraction prostate‐motion traces of (a) fraction number 10, proton irradiation started at 25th second; (b) fraction number 13, proton irradiation started at 25th second; and the faction DVH from the simulated treatments of (c) fraction number 10; (d) fraction number 13. DVH, dose‐volume histogram.

Figure 2 shows the prostate‐motion traces of two treatment fractions and their corresponding DVHs from simulated proton treatments for both DS and US deliveries. For comparison, the original Eclipse treatment plan DVHs are also presented. As evidenced, minimum prostate motion leads to almost identical CTV DVHs between that of the Eclipse plan and those of the simulated DS and US deliveries. When there was significant prostate motion, there was degradation of the CTV DVHs for both the DS and US deliveries. However, the DVH of simulated US treatment showed that there are more hot (overdose i.e. V110> 2%) and cold (underdose i.e. D95% < 95%) spots in the CTV. For the simulated DS treatment, there was mainly underdosing of the CTV.

**Figure 3:**
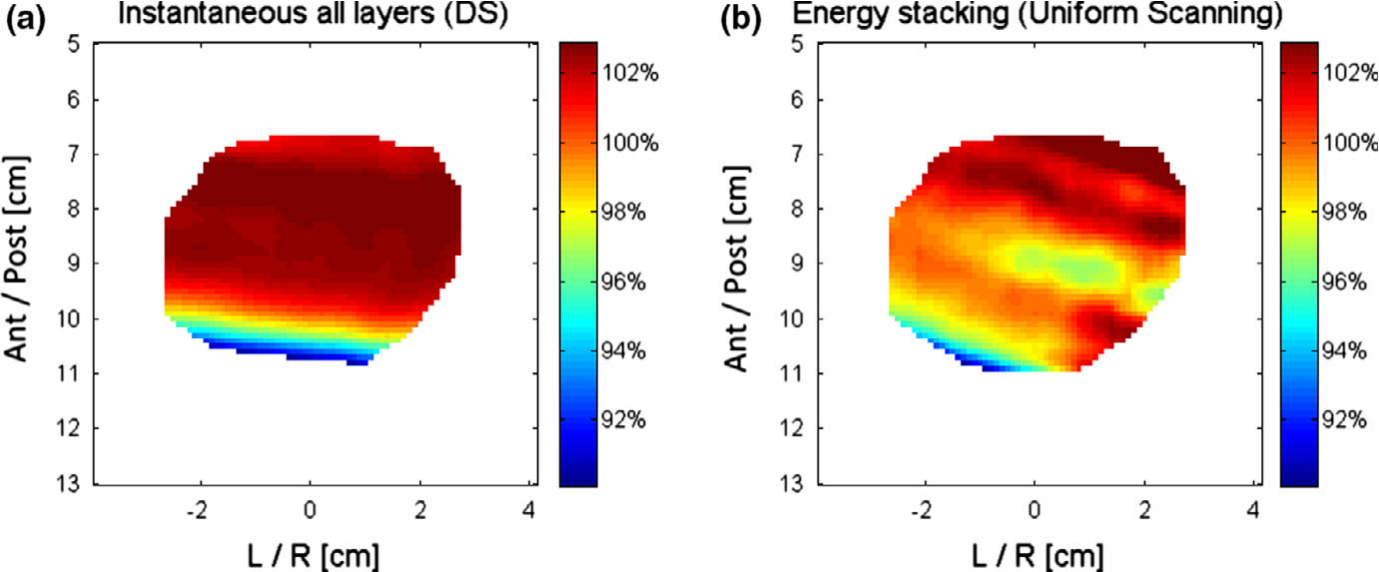
For a right‐anterior oblique beam in a single fraction delivery simulation, the planar dose in the transverse plane through the isocenter (a) in the case of double scattering delivery; (b) in case of uniform scanning delivery for the same intrafraction prostate motion.

**Figure 4:**
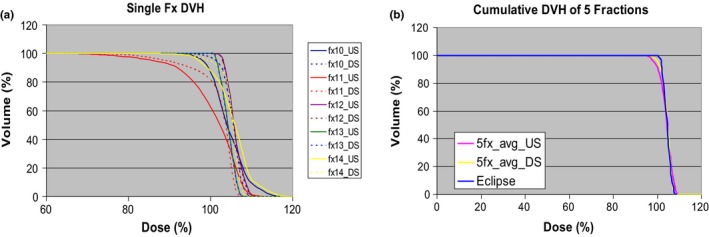
Clinical target volume dose‐volume histogram of (a) Five single fractions; (b) cumulative dose‐volume histogram for both DS and US. DS, double scattering; US, uniform scanning.

Figure 3 shows fraction dose distributions for both DS and US delivery with the same intrafraction prostate motion. The slightly inhomogeneous dose can be observed in the US dose distribution. Figure 4 shows CTV DVHs of five single fractions of simulated proton treatment as well as the DVHs of the sum of the five fractions for both DS and US deliveries. The individual‐fraction DVH clearly demonstrates the CTV DVH variation’s dependence on prostate intrafraction motion. Fraction 11 has the worst DVH of all the simulated patient treatment fractions for both DS and US deliveries. This demonstrated the largest dosimetric variation. Nevertheless, the DVHs of the 5‐fraction sum of simulated DS and US deliveries show only minor deviations from the DVH of the Eclipse treatment plan.

Table 1 shows CTV DVH points for all simulated individual treatment fractions for both DS and US deliveries. The mean values and maximum values (except V110) over 238 fractions of the DVH points are comparable between the DS and US deliveries. However, the maximum of V110, the minimum and standard deviation of the DVH point values in these fractions demonstrated the impact of prostate intrafraction motion on US deliveries. Systematically smaller minimum values of the DVH points for US deliveries indicated colder spots (more underdosed) inside CTV than those of DS deliveries. Greater maximum values of V110 for US deliveries indicated hotter spots (more overdosing) inside CTV than those of DS deliveries. Larger standard deviations of the DVH points for US deliveries indicated that there is larger CTV dosimetric uncertainties compared to DS deliveries with the same intrafraction prostate motion. However, student t‐test indicated that there are no statistically significant dose differences between the DS and US treatments except for values of V110. Regular‐fractionation prostate radiotherapy usually delivers treatment in more than 30 fractions. Some of the hot and cold spots for the CTV dose in individual fractions can be washed out after many fractions of treatment. Table 2 shows DVHs for the 14‐fraction sum for all 17 of the simulated patients. Compared to Table 1, there was significant improvement in the minimum values and standard deviation values for both DS and US deliveries. V110 has zero values for all the patient cumulative doses; this indicates that all the V110 hotspots are all washed out. V95 has 100 percent for all patients, indicating there is no cold spot below 95% of prescribed dose inside prostate CTV. Similar dosimetric trends can be observed in Table 3 and 4 from the SBRT simulations of the individual fraction and the cumulative 5 fraction treatment.

**Table 1:** Clinical Target Volume dose‐volume histogram percentage points for 238 single‐fraction dose and the *P*‐values of student t‐test between DS and US of 2 Gy per fraction simulations.

		V100	V95	V110	D100	D95	D5
Eclipse		99.9	100	0	99.6	101.8	107.3
Mean	DS	99.6	99.9	0.1	99.8	103.2	108.6
	US	99.1	99.8	0.4	99.5	103.1	108.8
STD	DS	1.8	0.7	0.2	3.9	1.2	1.2
	US	3.7	1.3	1.2	4.4	1.7	1.3
Max	DS	100.0	100.0	0.4	101.0	104.7	109.9
	US	100.0	100.0	12.2	102.0	104.8	114.6
Min	DS	81.5	90.3	0.0	67.0	90.6	106.6
	US	62.6	82.8	0.0	61.0	87.5	106.8
*P* value		0.052	0.430	0.001	0.412	0.297	0.113

DS, double scattering; STD, standard deviation; US, uniform scanning.

**Table 2:** Clinical Target Volume cumulative dose‐volume histogram percentage points of all 17 patients and the *P*‐values of student t‐test between DS and US of the 2 Gy per fraction simulations.

		V100	V95	V110	D100	D95	D5
Eclipse		99.9	100	0	99.6	101.8	107.3
Mean	DS	99.9	100.0	0.0	100.6	103.6	108.3
	US	99.9	100.0	0.0	100.6	103.6	108.3
STD	DS	0.2	0.0	0.0	1.1	0.2	0.2
	US	0.3	0.0	0.0	1.3	0.2	0.2
Max	DS	100.0	100.0	0.0	101.0	103.7	108.4
	US	100.0	100.0	0.0	102.0	103.8	108.5
Min	DS	99.3	100.0	0.0	97.0	102.8	107.9
	US	98.8	100.0	0.0	96.0	102.7	107.7
*p* value		0.721	N/A	N/A	1.0	0.940	0.650

DS, double scattering; N/A, not available; STD, standard deviation; US, uniform scanning.

**Table 3:** Clinical Target Volume dose‐volume histogram percentage points of 85 single‐faction dose and the *P*‐values of student t‐test between DS and US of the SBRT simulations.

		V100	V95	V110	D100	D95	D5
Eclipse		99.9	100	0	99.6	101.8	107.3
Mean	DS	98.6	99.5	0.1	97.5	102.5	108.3
	US	97.7	99.4	0.3	96.9	102.4	108.6
STD	DS	4.1	2	0.1	9	2.7	1.2
	US	5.9	2.1	0.7	9.3	2.9	1.2
Max	DS	100.0	100.0	0.4	101.0	104.6	109.9
	US	100.0	100.0	3.8	101.0	104.7	110.6
Min	DS	79	89.5	0.0	60	90.6	106.4
	US	70.3	90.4	0.0	60.0	91.7	106.7
*P* value		0.301	0.885	0.003	0.727	0.842	0.105

DS, double scattering; STD, standard deviation; US, uniform scanning.

**Table 4:** Clinical Target Volume cumulative dose‐volume histogram percentage points of all 17 patients and the *P*‐values of student t‐test between DS and US of the SBRT simulations.

		V100	V95	V110	D100	D95	D5
Eclipse		99.9	100	0	99.6	101.8	107.3
Mean	DS	98.8	99.7	0.0	98.9	103.1	108.0
	US	98.7	99.7	0.0	98.9	103.1	108.1
STD	DS	4.0	1.1	0.0	5.2	0.2	0.4
	US	4.2	1.2	0.0	5.1	0.3	0.4
Max	DS	100.0	100.0	0.0	101.0	103.7	108.2
	US	100.0	100.0	0.0	101.0	103.8	108.5
Min	DS	85.1	96.1	0.0	82	97	107.0
	US	84.3	95.7	0.0	82	96.7	107.1
*P* value		0.963	0.946	N/A	1.0	0.967	0.656

DS, double scattering; N/A, not available; STD, standard deviation; US, uniform scanning.

## 4 DISCUSSION

Many investigators have studied target intrafraction motion and its dosimetric impact on CTV dose coverage in photon and proton radiation therapy.^15^, ^16^, ^17^, ^18^, ^19^, ^20^ This study focused on the simulation of DS and US proton treatment using real patients’ prostate intrafraction motion traces and relevant clinical treatment plans. The results of this study not only provide an insight into the real dosimetric impacts of prostate intrafraction motion on proton treatment with both DS and US techniques, they also reveal the effects of the interplay between sequential energy‐layer delivery of US and temporal prostate motion.

This study indicated that, the majority of prostate intrafraction motions usually cause minimal CTV dose degradation in both DS and US delivery modes. When there was significant intrafraction motion in DS delivery, there was usually CTV underdosage, which manifested into a broad shoulder in the CTV DVH. For the same intrafraction motion in the beam axis direction in US delivery, the spatial‐temporal interplay between the individual energy‐layer delivery and prostate motion can lead to over‐irradiation or under‐irradiation of the prostate. Thus, the CTV dose inhomogeneity was greater in the US delivery than in the DS delivery with the same intrafraction motion traces.

For the prostate‐motion traces used in this study, the predominant intrafraction motion was in the superior‐inferior and anterior‐posterior directions. The left‐right direction motion was the smallest in magnitude and frequency in all three axes. At our institution, almost all prostate‐patient treatment fields are either lateral or lateral oblique (within 10 degrees of the lateral direction). Therefore, the effect of the spatial‐temporal interplay in the US delivery is mainly caused by prostate motion in the lateral direction. Prostate motion in the anterior‐posterior and superior‐inferior directions affect both DS and US deliveries in a similar way that potentially underdose the periphery of the CTV, depending on the magnitude of the motion. For US delivery, the timing of prostate motion is also one of the factors influencing the CTV DVH. Generally, large magnitudes of prostate motion that occur in initial (distal) several layers would have a large CTV dosimetric impact due to their large dose weightings in the generation of SOBPs.

Even though the proton treatment simulation incorporated the prostate motion and energy layer delivery timing to investigate the impact of intrafraction motion on prostate CTV dosimetry, not all the details of the US delivery were incorporated in the simulation. For example, the scanning frequencies of 3 Hz in the gantry rotation axis direction and 30 Hz in its orthogonal direction as well as the repainting of each energy layer were not simulated. We believe that rapid‐beam spot scanning and repainting of each energy layer equivalently created a quasi‐instantaneous dose cloud for each energy layer. Thus, in US delivery simulation, moving the prostate through the dose matrix of each energy layer based on its time weight is dosimetrically adequate and accurate.

## 5 CONCLUSION

In this study, we simulated both DS and US proton treatment of prostate using real‐patient prostate‐motion traces and evaluated dosimetric impact of intrafraction motion on both delivery techniques. The fraction dose analyses indicated that CTV dose degradation due to prostate intrafraction motion is patient and fraction specific. Severe intrafraction prostate motion can cause CTV hot and cold spots in US treatments, whereas it only causes CTV underdosing in DS treatments. However, no statistically significant dose differences were observed between the two treatment delivery techniques. The cumulative dose of several simulated treatment fractions showed that the magnitude of the CTV dose degradation was reduced and generally lies within a clinically acceptable range from planned dose distributions. Nevertheless, the effects of target intrafraction motion can be a concern for other, more dynamic targets.

## CONFLICT OF INTEREST

No conflicts of interest.
